# Nonassortative relationships between groups of nodes are typical in complex networks

**DOI:** 10.1093/pnasnexus/pgad364

**Published:** 2023-11-06

**Authors:** Cathy Xuanchi Liu, Tristram J Alexander, Eduardo G Altmann

**Affiliations:** School of Mathematics and Statistics, University of Sydney, Sydney, 2006 NSW, Australia; Centre for Complex Systems, University of Sydney, Sydney, 2006 NSW, Australia; Centre for Complex Systems, University of Sydney, Sydney, 2006 NSW, Australia; School of Physics, University of Sydney, Sydney, 2006 NSW, Australia; School of Mathematics and Statistics, University of Sydney, Sydney, 2006 NSW, Australia; Centre for Complex Systems, University of Sydney, Sydney, 2006 NSW, Australia

## Abstract

Decomposing a graph into groups of nodes that share similar connectivity properties is essential to understand the organization and function of complex networks. Previous works have focused on groups with specific relationships between group members, such as assortative communities or core–periphery structures, developing computational methods to find these mesoscale structures within a network. Here, we go beyond these two traditional cases and introduce a methodology that is able to identify and systematically classify all possible community types in directed multi graphs, based on the pairwise relationship between groups. We apply our approach to 53 different networks and find that assortative communities are the most common structures, but that previously unexplored types appear in almost every network. A particularly prevalent new type of relationship, which we call a *source–basin* structure, has information flowing from a sparsely connected group of nodes (source) to a densely connected group (basin). We look in detail at two online social networks—a new network of Twitter users and a well-studied network of political blogs—and find that source–basin structures play an important role in both of them. This confirms not only the widespread appearance of nonassortative structures but also the potential of hitherto unidentified relationships to explain the organization of complex networks.

**Significance Statement**

Networks are a powerful mathematical representation of various datasets and systems. An important computational tool to study and extract information from large complex networks is to partition it into groups of nodes with similar connectivity. Previous works focused on groups forming so-called assortative communities, in which nodes link preferentially to other nodes in the same group. In our work, we show that other (previously unexplored) types of community organizations are ubiquituous in complex networks. In particular, we find that a new type of “source–basin” structure organizes the flow of information in online social networks, which happens from a community of sparsely connected influential nodes (the source) to a community of densely inter-connected active nodes (the basin).

## Introduction

An important step in the analysis of complex networks is the partitioning of nodes into groups or communities according to their connectivity pattern (1–5). Most approaches focus on finding groups in which users link preferentially to other users of the same group. A variety of computational methods have been proposed to identify such *assortative communities* (2, 3, 6, 7), such as, modularity maximization (8, 9), spectral methods (10), and Infomap (11). Another well-studied form of network organization is the *core–periphery structure*, in which information flows from a tightly connected core community of users to a loosely connected periphery (12–14). Here, also different algorithms have been specifically proposed to identify such structures (12, 15–17).

More recent research has shown the importance of going beyond these “descriptive methods,” which seek predefined structures, and instead use “inferential methods” (18–20) that can learn the most relevant structure from the data. These methods are robust against the detection of spurious communities (e.g. communities in simple random graphs), and provide better partitions of the graph in groups (in terms of better compression of data and retrieval of partitions in synthetic networks) (19–21). Inferential approaches connect the network-partition problem to generative models and random-network ensembles, settings in which a richer variety of structures appear naturally (22, 23). In particular, inferential methods based on stochastic block models (SBM) (24–26) do not commit to specific types of community structures (e.g. assortative or core–periphery), leaving it to the data to determine the most significant statistical signature that leads to the clustering of nodes into blocks (communities). In view of this key advantage of modern community-detection methods, several natural questions arise: to what extent are assortative or core–periphery structures dominant in networks? Are there other types of community structures that were not previously investigated? If so, what do these structures reveal about the organization and function of complex networks?

**Fig. 1.**
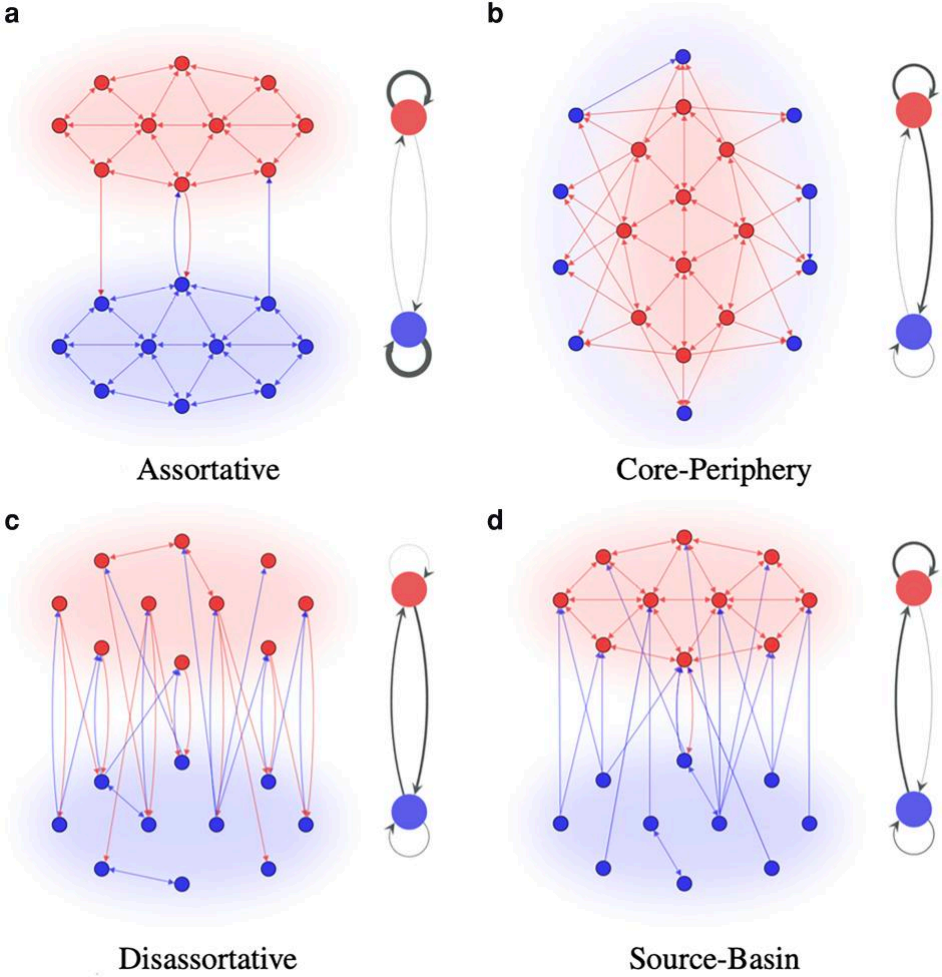
The four main types of community relationship in directed graphs: assortative a), core–periphery b), disassortative c), and source–basin d). The left side corresponds to the graph with nodes colored according to their community assignment. The right side is a graphical representation of the edge density matrix ω for the two communities.

In this paper, we answer these questions by introducing a systematic classification of community structures in undirected and directed multigraphs. Figure 1 illustrates the four types of structure present at the highest level of our classification in the directed case. Our analysis of different network data, sampling across both directed and undirected networks, reveals that assortative relationships are the most prevalent type of relationship between communities—being the dominant feature in at least 69% of networks—but that relationships that are neither assortative nor core–periphery appear in 68% of the networks. Our detailed analysis of two case studies—the widely studied political blogs network (27) and one novel network of Twitter users (28)—confirms that new types of communities play an important role in the organization of online social networks.

## Methodology

The community-detection problem in networks corresponds to partitioning a graph *g* (possibly directed and weighted) with adjacency matrix $A_{ij}\in N$ into r=1,2,…,B disjoint groups with $N_{r}$ nodes. For a given community partition of $A_{ij}$, we characterize the connection between a pair of communities *r* and *s* based on their density $\omega_{rs}$ which is given by the ratio of existing links and possible links:


(1) $$\omega_{rs}=\left\{ \begin{array}{ll} \sum_{i\in r,j\in s}\frac{A_{ij}}{N_{r}N_{s}},\; & \text{if }r\neq s\\ \sum_{i\in r,j\in s}\frac{A_{ij}}{\frac{1}{2}N_{r}(N_{s}-1)},\; & \text{if }r=s \end{array}\right.$$

for undirected graphs ($A_{ij}=A_{ji}$) and


(2) $$\omega_{rs}=\left\{ \begin{array}{ll} \sum_{i\in r,j\in s}\frac{A_{ij}}{N_{r}N_{s}},\; & \text{if }r\neq s\\ \sum_{i\in r,j\in s}\frac{A_{ij}}{N_{r}(N_{s}-1)},\; & \text{if }r=s \end{array}\right.$$

for directed graphs. Without loss of generality, we order the label of communities so that $\omega_{rr}\geq \omega_{ss}$ for r<s. Each community-detection method applied to a network *g* will typically provide a different B×B density matrix ω and our methodology developed below applies to any method.

**Fig. 2.**
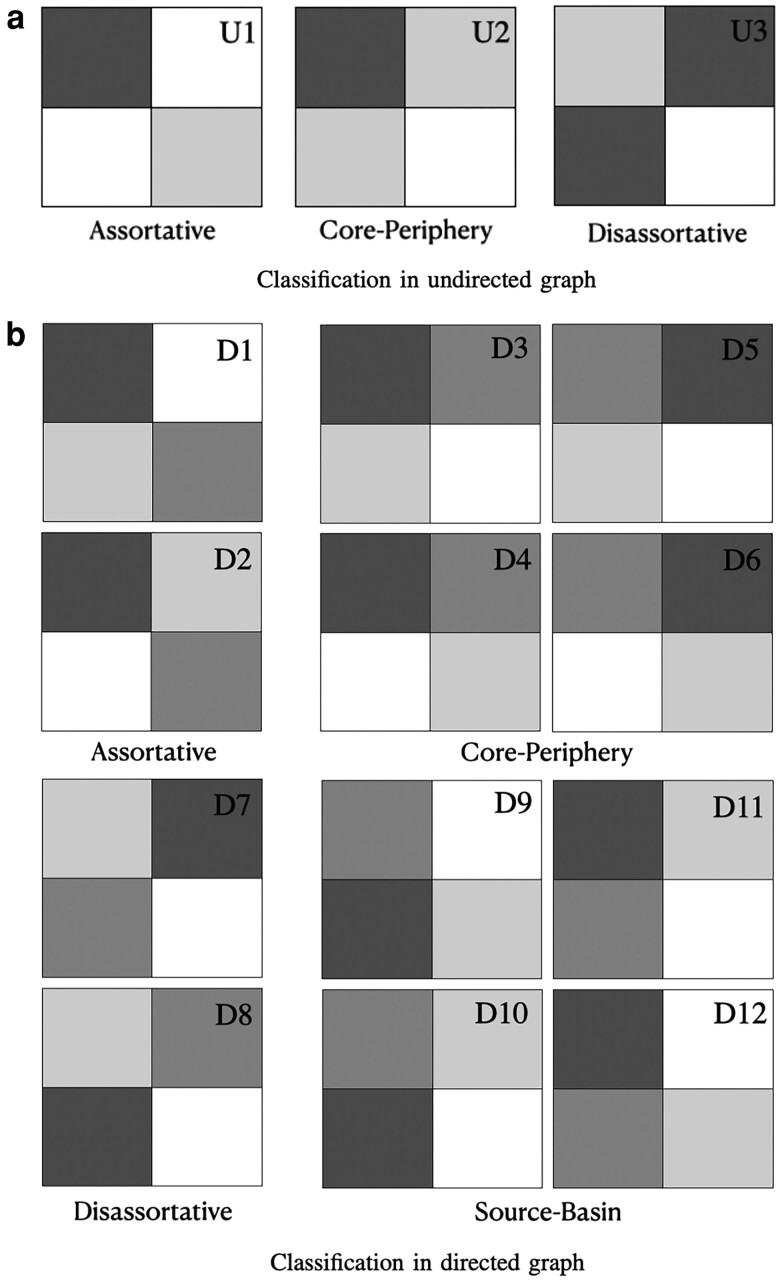
Community type classification. a) The 3 possible community types in graphs with undirected edges. b) The 12 different possible configurations in directed graphs classified in four types. We use the convention that the link direction corresponds to the flow of information (or influence) so that a link from node *i* to *j* indicates that information is passing from *i* to *j* (or that *i* influences *j*). Assortative (disassortative) cases have the two largest entries in the diagonal $\omega_{rr},\omega_{ss}$ (antidiagonal $\omega_{rs},\omega_{sr}$). The remaining eight cases have a densely connected community (top), in which nodes are well connected to each other, and a loosely connected community (bottom). In four core–periphery cases, the influence flows from the well-connected core to the weakly connected periphery, while in the remaining four cases it flows in the reverse direction. We name this reversed core–periphery structure a source–basin structure.

We now classify the relationship between a pair of communities *r* and *s* based on $\omega_{rs}$. In the simplest case of undirected graphs *g*, there are three relevant quantities $\omega_{rr},\omega_{ss},$ and $\omega_{rs}=\omega_{sr}$. Ranking these values by size, there are three possible configurations U1, U2, and U3 (13, 29), as illustrated in Fig. 2a. They correspond to the two previously studied types of community structures—assortative and core–periphery—and a new case—disassortative—that has strong connections between communities (the limiting scenario $\omega_{rr}=\omega_{ss}=0$ corresponds to a bi-partite network). A categorical classification of the pairwise interaction between communities *r* and *s* based on the ranking order of the $\omega_{rs}$ values can be written as


(3) $$\left\{ \begin{array}{ll} \text{Assortative}, & \text{if }\omega_{rs}<\min\left(\omega_{rr},\omega_{ss}\right),\\ \text{Core}-\text{periphery}, & \text{if }\min\left(\omega_{rr},\omega_{ss}\right)<\omega_{rs}<\max\left(\omega_{rr},\omega_{ss}\right),\\ \text{Disassortative}, & \text{if }\omega_{rs}>\max\left(\omega_{rr},\omega_{ss}\right). \end{array}\right.$$

The case for directed graphs *g* leads to a wider variety of possible configurations between communities *r* and *s*. Following the same approach employed for undirected graphs, we obtain 12 possible configurations D1,D2,…,D12 based on the ranking of the four quantities $\omega_{rr},\omega_{rs},\omega_{sr},$ and $\omega_{ss}$. Figure 2b shows these 12 cases, grouped into 4 types. Configurations with the same general interpretation are grouped into the same type, which can be one of the three types observed in undirected graphs (assortative, core–periphery, and disassortative) or one new type. In particular, in the new type of relationship—denoted source–basin—influence flows from loosely connected source nodes to a basin of inter-connected nodes. Contrary to the core–periphery relationship, the most influential and central nodes (in the source community) do not form a core as they are more connected to nodes in the other community (the basin) than to nodes in their own community.

The categorical classification of the pairwise interaction between communities *r* and *s* into different community structure types based on ω for a directed graph can be written as


(4) $$\left\{ \begin{array}{ll} \text{Assortative}, & \text{if }\max\left(\omega_{rs},\omega_{sr}\right)<\min\left(\omega_{rr},\omega_{ss}\right),\\ \text{Core}-\text{periphery}, & \text{if }\min\left(\omega_{rr},\omega_{rs}\right)>\max\left(\omega_{sr},\omega_{ss}\right),\\ \text{Disassortative}, & \text{if }\min\left(\omega_{rs},\omega_{sr}\right)>\max\left(\omega_{rr},\omega_{ss}\right),\\ \text{Source}-\text{basin}, & \text{if }\min\left(\omega_{rr},\omega_{sr}\right)>\max\left(\omega_{rs},\omega_{ss}\right). \end{array}\right.$$

The assortative (disassortative) interaction is thus defined naturally as having more links to other nodes in the same (other) communities. Core–periphery and source–basin are defined as interactions that show a mixed behavior, with one sparser community more linked to the other community and one denser community more linked to itself. The difference between the two types of interactions is that in the core–periphery the links (influence/information) across communities flow preferentially from the denser to the sparser community, while in the source–basin interaction the flow is in the reversed direction.

**Fig. 3.**
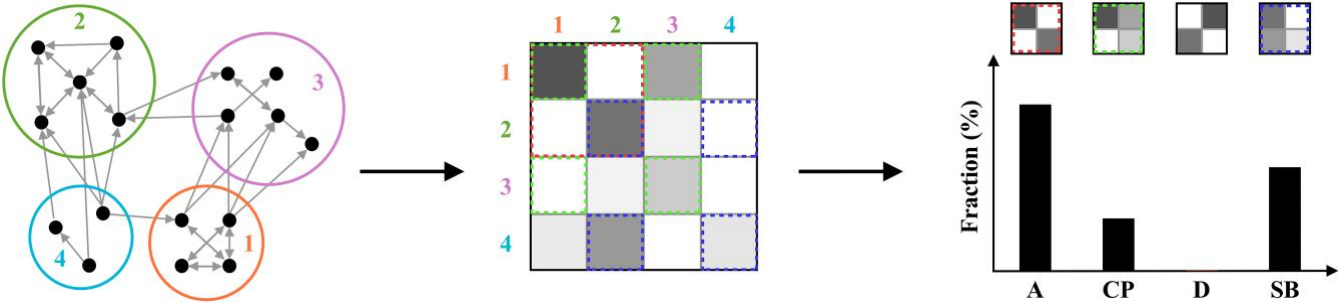
Illustration of our methodology for classifying community types. (Left) Applying a given community-detection method to a network *g*, we obtain a partition of the nodes in *B* communities (B=4 in the example). (Center) From the node partition we construct the B×B density matrix ω from Eq. 1 or 2. (Right) We apply the pairwise classification defined in Fig. 2—and Eqs. 3 and 4—to ω considering all pairwise combinations of communities r,s and compute the fraction of each of the four different types of community relationships, where “A” stands for “assortative,” “CP” stands for “core–periphery,” “D” stands for “disassortative” and “SB” stands for “source–basin.”

The final step of our methodology is to apply the pairwise classification we just introduced to networks containing B>2 communities. Our approach is to look at each of the possible pairwise interactions, classify its structure type τ∈ {“assortative,” “core–periphery,” “disassortative,” “source–basin”} and measure the prevalence of different community structure types, as described in Fig. 3.

We test the methodology proposed above applying five community-detection methods to 52 real-world networks. The networks were retrieved from the Netzschleuder repository (30) and were selected to include both directed (26) and undirected (26) networks from a variety of domains and sizes (from 75 to 14,360 vertices and from 181 to 150,985 edges, see Supplementary Section S1 for details). The five community-detection methods—Louvain (modularity maximization) (9, 31), Infomap (11, 32), Spectral method (33, 34), degree-corrected SBM (26, 35), and deep neural networks for graph representations (DNGR) (36, 37)—were selected as representative of different approaches to this problem [see Supplementary Section S2 for details and our repository (38) for the numerical implementation]. As pointed out in the Introduction section, we are particularly interested in inference-based methods (39), in particular degree-corrected SBM, because of their weaker assumptions on the possible structure of ω.

**Fig. 4.**
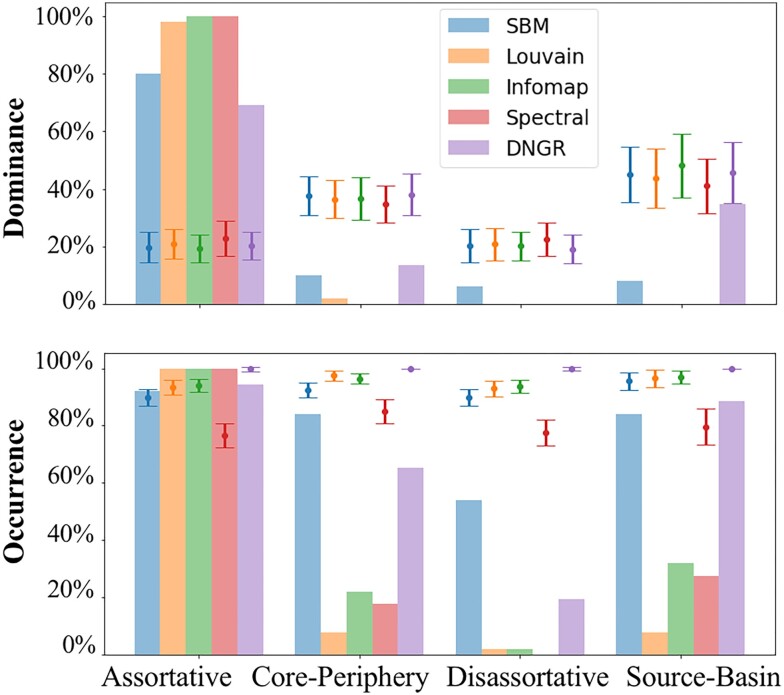
Community types in empirical directed and undirected networks. Top: fraction of networks (*y*-axis) in which each community type (*x*-axis) is dominant. Bottom: fraction of networks (*y*-axis) in which each community type (*x*-axis) occurs. For the source–basin classification, only directed networks are considered. See Supplementary Section S2 for details on the community-detection methods (listed in the legend) and Supplementary Section S3 for details on the classification of the results. The symbols with error bars were obtained in a null-model which considers a random edge-density matrix ω (see Supplementary Section S4 for details).

The results summarized in Fig. 4, averaging across both directed and undirected networks, show that assortative relationships are the dominant relationship type in most networks for all methods. In the three methods that target such structures (Spectral, Infomap, and Louvain), it is the dominant structure in almost all cases (one exception of Louvain is found for a network with only three communities detected, see Supplementary Fig. S1 for details) and there are in fact very few networks in which nonassortative structures are detected. More interestingly, the SBM method found nonassortative community relationships in a large fraction of the networks (92%). This corresponds not only to the well-studied core–periphery relationship (84%) but also the disassortative (54%) and the intriguing source–basin relationship (81% of directed graphs). A diverse distribution is also found using the DNGR method, with an even stronger presence of the source–basin relationshop (92%). Similar results are observed when we restrict the analysis to only undirected or directed networks, or compute the average fraction of different community types, see Supplementary Section S2. Overall, this finding demonstrates that nonassortative communities are not only a theoretical possibility, they are typical in empirical networks, as long as one uses methods that allow for this possibility and do not focus exclusively on finding assortative partitions. These results confirm also the practical importance of the theoretical advantages of inferential methods such as SBM, in particular of not being restricted to specific community structures.

**Fig. 5.**
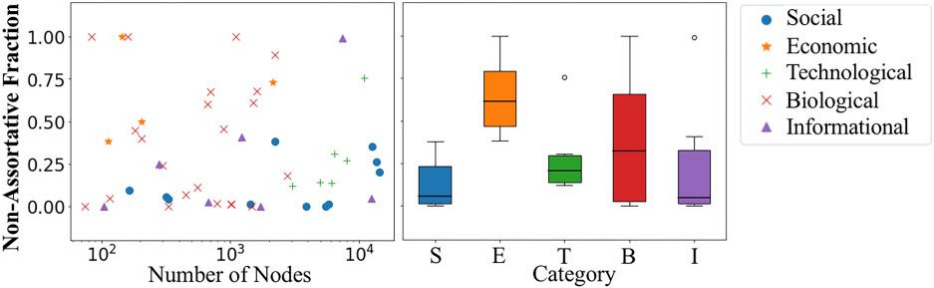
Nonassortative relationships are found in a variety of empirical directed and undirected networks. Left: fraction of nonassortative relationship (*y*-axis) as a function of the number of nodes (*x*-axis), with each symbol representing a network (see legend for their categories). Right: Boxplot of nonassortative fraction for networks grouped into their categories: Social (S), Economic (E), Technological (T), Biological (B), and Informational (I). The results correspond to the communities obtained using the SBM method.

Given the flexibility of SBM, including the method’s freedom to discover an optimal number of communities, we focus on this method and compare results obtained in networks from five different domains. Figure 5 shows that economic and biological networks have overall higher nonassortative fraction—with median fraction larger than 0.25 and higher variability—while social, technological, and informational networks have fewer nonassortative interactions. In particular, the fact that SBM found such arrangements indicates that they provide a more plausible partition of the nodes into communities than any partition that does not involve them. In the next section, we obtain further insights on the interpretation and significance of source–basin structures through the detailed study of two particular cases.

## Case studies

We consider two case studies that aim to understand the significance and interpretation of the unusual, nonassortative, community relationships that we found to be common in the previous section. We focus on the SBM method because of its ability to detect such patterns. We use a degree-corrected SBM (25) so that the community allocation of nodes is not dominated by their number of links (degree) but instead by how these links are distributed across other communities. We increase the number of blocks *B* from two until we detect the first nonassortative communities because we are interested in the simplest settings in which they appear.

**Fig. 6.**
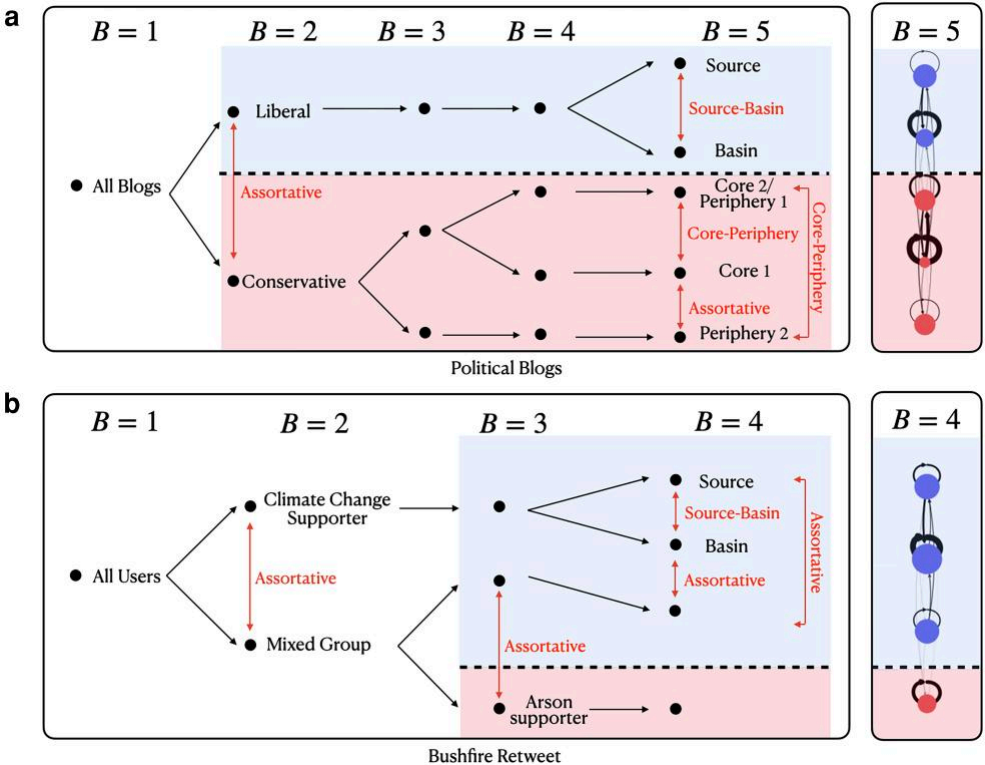
New types of community structures are typical in online social networks. Affiliation separation in a) the political blog network (27) and b) the bushfire retweet network (28). The partition into groups was obtained using the degree-corrected SBM method with increasing number of communities *B*. The main plot shows schematically how communities divide and the type of pairwise interaction they share (communities at larger *B* typically have the same interactions as their parent communities at lower *B* so that only new interactions are indicated). The plot at the right shows for the largest *B* the graphical representation of the density matrix ω (see Supplementary Fig. S2 for the complete classification). The labels in the plots (starting at B=2) are based on the manually classification of the political stance of users (in all labeled and colored groups, more than 73% of the classified users shared the same classification).

The first case we consider is the famous blog network introduced in Ref. (27). Each node corresponds to a political blog active during the US Presidential Election of 2004 and each directed edge j↦i corresponds to a hyperlink from blog *i* to blog *j* (this choice of edge direction corresponds to our convention of aligning it to influence or information flow). Nodes have been self-reported or manually tagged as “liberal” and “conservatives,” depending on the alignment to the two main parties in United States of America’s political election in 2004. This network has been extensively studied, e.g. the retrieval of the two groups has often been used as a benchmark in the development of new community-detection methods. Importantly, only assortative and core–periphery communities have been reported (17, 26), in line with the limitations of traditional community-detection methods which target exclusively these types of structures. In Fig. 6a, we show that our more general approach finds source–basin structures as a statistically significant partition of nodes already for B=5. This analysis reveals a fundamental difference between the liberal and conservative blogosphere, with conservative blogs organized internally in assortative and core–periphery structures, while liberal blogs show the unusual source–basin structure. Looking at the 10 most referenced blogs (top in out-degree), 4 belong to the source community and none to the basin community. This surprising finding for the liberal blogosphere suggests that the information-source blogs (being heavily referenced) are not tightly connected with each other, while information-receiver blogs are less referenced by others but highly active in referencing others and strongly connected with each other.

Having shown the appearance of new community structures in a well-known network, we now focus on a social-media example obtained following practices in computational social science (see Supplementary Section S1). The nodes in the resulting network correspond to 4,029 Twitter users that were influential in the political debate around the role of climate change in the severe bushfires in Australia in 2019–2020. The directed links i↦j reflect user *j* retweeting a message originating with user *i* (information flows from *i* to *j*). A fraction of the most influential messages were manually tagged as supporting or rejecting (1) a link between the bushfires and climate or (2) a link between the bushfires and the action of arsonists (28). This allows us to identify users that support the causal connection between climate change and the bushfires and those that deny it in favor of the (unsubstantiated) claim that the fires were due to arsonists. Our community-detection results shown in Fig. 6b confirm that the separation of these users into assortative communities is a dominant feature of the network, and corroborates the significance of our approach (which is not based on content). Beyond this expected result, we find that already with B=4 communities there are nonassortative relationships within the major assortative separation. Looking at the users in these communities, we find that the source community contains high-profile users (9 of the top-10 most retweeted users, 31% of the users are verified) who retweet more rarely (average in-degree is 99). In contrast, the basin community has fewer high-profile users (0 of the top-10 most retweeted users, only 7% of the users are verified) and they are more active in the debate (average in-degree is 225).

Our finding and analysis of the source–basin structures in both cases discussed above suggests a new picture for the flow of information in online networks. The group that act as a source of information (within one of the sides of the polarized political debate) contains the more central (high-status) nodes which do not interact significantly with each other. This possibly happens because the source users compete for the attention of the basin users within their side of the political division (i.e. on the same side of the main assortative division of the network). In the basin community, the receivers are more strongly connected with each other, discussing and referencing both source nodes and each other during the debate.

## Conclusion

While mesoscale structures in complex networks have been successfully explored using community-detection methods for more than two decades, the majority of studies assume communities to have either assortative or core–periphery connectivity patterns. As we have shown, this does not exhaust the possible connectivity patterns between communities. The main finding of this work is to show that these additional patterns are not only possible, they are typical—as long as communities are detected using methods that are agnostic about the underlying type of communities—and important to understand the underlying complex system. In particular, we find a new community arrangement in which information (influence) flows from a source community of nodes—weakly connected with each other—toward a basin community of information receivers that engage repeatedly with each other forming a strongly connected community. This “source–basin” structure reveals a new type of organization of social-media networks that has evaded previous analysis (mostly based on methods that target specific structures) and that is typical also in other networks. In fact, our survey of 52 networks shows that nonassortative community structures appear in all categories of networks, being more prevalent in economic and biological networks and less prevalent in social, technological, and informational networks.

The methodology we introduced to identify and quantify the prevalence of unusual communities in networks can be applied to any directed multigraphs. Evidence of its potential to reveal new insights is that it found unusual community structures in both a paradigmatic network in community-detection studies—the political blogs network—and in a new social-media network constructed through standard procedures in computational social science. The fact that such community patterns have not been previously reported in these cases, despite being extensively studied networks and construction approaches, confirms both the potential of our methodology and the limitations of community-detection methods that fix a priori specific structures. We can thus expect that studies with networks in other areas will be similarly successful in identifying unusual structures and providing new insights not only on the role of specific nodes and communities but also on the organization and function of the network as a whole.

Beyond applications to specific settings, future work should consider how to extend the applicability and interpretability of our methods and findings. For instance, generalizations that overcome simplifying assumptions of our methods could consider more general types networks (e.g. multilayered) and definitions of community type (e.g. beyond pairwise classification). To better understand the findings, a complimentary approach to our data-driven methods is to consider generative models that give rise to the different community types we detect. A case of special interest is to reveal mechanisms that lead to source–basin structures.

## Supplementary Material

pgad364_Supplementary_Data

## Data Availability

The data used in our survey is available in the Netzschleuder repository (30). The Twitter-user network used in our case study is available in our repository (38), together with all codes needed to reproduce our results and apply our methodology to other networks.

## References

[1] Newman M . 2018. Networks. Oxford: Oxford University Press.

[2] Guimera R, Sales-Pardo M, Amaral LAN. 2007. Classes of complex networks defined by role-to-role connectivity profiles. Nat Phys. 3(1):63–69. 18618010 10.1038/nphys489 PMC2447920

[3] Fortunato S . 2010. Community detection in graphs. Phys Rep. 486(3–5):75–174.

[4] Fortunato S, Newman MEJ. 2022. 20 years of network community detection. Nat Phys. 18(8):848–850.

[5] Liu F , *et al*. 2020. Deep learning for community detection: progress, challenges and opportunities. Proceedings of the Twenty-Ninth International Joint Conference on Artificial Intelligence, IJCAI-20: 4982, January 7–15, 2021, Yokohama, Japan.

[6] Radicchi F, Castellano C, Cecconi F, Loreto V, Parisi D. 2004. Defining and identifying communities in networks. Proc Natl Acad Sci USA. 101(9):2658–2663. 14981240 10.1073/pnas.0400054101 PMC365677

[7] Danon L, Diaz-Guilera A, Duch J, Arenas A. 2005. Comparing community structure identification. J Stat Mech Theory Exp. 2005(09):P09008.

[8] Clauset A, Newman MEJ, Moore C. 2004. Finding community structure in very large networks. Phys Rev E. 70(6):066111. 10.1103/PhysRevE.70.066111 15697438

[9] Blondel VD, Guillaume J-L, Lambiotte R, Lefebvre E. 2008. Fast unfolding of communities in large networks. J Stat Mech Theory Exp. 2008(10):P10008.

[10] Von Luxburg U . 2007. A tutorial on spectral clustering. Stat Comput. 17:395–416.

[11] Rosvall M, Bergstrom CT. 2008. Maps of random walks on complex networks reveal community structure. Proc Natl Acad Sci USA. 105(4):1118–1123. 18216267 10.1073/pnas.0706851105 PMC2234100

[12] Borgatti SP, Everett MG. 2000. Models of core/periphery structures. Soc Networks. 21(4):375–395.

[13] Zhang X, Martin T, Newman MEJ. 2015. Identification of core–periphery structure in networks. Phys Rev E. 91(3):032803. 10.1103/PhysRevE.91.032803 25871153

[14] Yang J, Zhang M, Shen KN, Ju X, Guo X. 2018. Structural correlation between communities and core–periphery structures in social networks: evidence from twitter data. Expert Syst Appl. 111:91–99.

[15] Rombach P, Porter MA, Fowler JH, Mucha PJ. 2017. Core–periphery structure in networks (revisited). SIAM Rev. 59(3):619–646.

[16] Elliott A, Chiu A, Bazzi M, Reinert G, Cucuringu M. 2020. Core–periphery structure in directed networks. Proc R Soc A. 476(2241):20190783. 33061788 10.1098/rspa.2019.0783 PMC7544362

[17] Kojaku S, Masuda N. 2017. Finding multiple core–periphery pairs in networks. Phys Rev E. 96(5):052313. 29347658 10.1103/PhysRevE.96.052313

[18] Peel L, Peixoto TP, De Domenico M. 2022. Statistical inference links data and theory in network science. Nat Commun. 13(1):6794. 36357376 10.1038/s41467-022-34267-9 PMC9649740

[19] Peixoto TP . 2023. Descriptive vs. inferential community detection in networks: pitfalls, myths, and half-truths. Elements in the structure and dynamics of complex networks. Cambridge: Cambridge University Press.

[20] Guimerà R . 2020. One model to rule them all in network science? Proc Natl Acad Sci USA. 117(41):25195–25197. 32989129 10.1073/pnas.2017807117 PMC7568293

[21] Ghasemian A, Hosseinmardi H, Clauset A. 2019. Evaluating overfit and underfit in models of network community structure. IEEE Trans Knowl Data Eng. 32(9):1722–1735.

[22] Bianconi G . 2007. The entropy of randomized network ensembles. Europhys Lett. 81(2):28005.

[23] Bianconi G, Pin P, Marsili M. 2009. Assessing the relevance of node features for network structure. Proc Natl Acad Sci USA. 106(28):11433–11438. 19571013 10.1073/pnas.0811511106 PMC2704854

[24] Holland PW, Blackmond Laskey K, Leinhardt S. 1983. Stochastic block models: first steps. Soc Networks. 5(2):109–137.

[25] Karrer B, Newman MEJ. 2011. Stochastic blockmodels and community structure in networks. Phys Rev E. 83(1):016107. 10.1103/PhysRevE.83.016107 21405744

[26] Peixoto TP . 2019. Bayesian stochastic blockmodeling. In: Doreian P, Batagelj V, Ferligoj A, editors. Advances in network clustering and blockmodeling. Hoboken (NJ): Wiley. p. 289–332.

[27] Adamic LA, Glance N. 2005. The political blogosphere and the 2004 US election: divided they blog. Proceedings of the 3rd International Workshop on Link Discovery. New York (NY): Association for Computing Machinery. p. 36–43.

[28] Bednarek M , *et al*. 2022. Winning the discursive struggle? The impact of a significant environmental crisis event on dominant climate discourses on twitter. Discourse Context Media. 45:100564.

[29] Betzel RF, Bertolero MA, Bassett DS. 2018. Non-assortative community structure in resting and task-evoked functional brain networks. bioRxiv 355016. 10.1101/355016.

[30] Peixoto TP . The netzschleuder network catalogue and repository [accessed 2022 Aug]. 10.5281/zenodo.7839980.

[31] Aynaud T . 2020. Python-Louvain x.y: Louvain algorithm for community detection [accessed 2021 Oct]. https://github.com/taynaud/python-louvain.

[32] Edler MRD, Eriksson A. 2020. Infomap network clustering algorithm [accessed 2022 Apr]. https://pypi.org/project/infomap/.

[33] Dall’Amico L, Couillet R, Tremblay N. 2019. Revisiting the Bethe-Hessian: improved community detection in sparse heterogeneous graphs. 33rd Conference on Neural Information Processing Systems (NeurIPS 2019), Vancouver, Canada.

[34] Dall’Amico L, Couillet R, Tremblay N. 2019. Revisiting the Bethe-Hessian: improved community detection in sparse heterogeneous graphs [accessed 2022 Sep]. https://lorenzodallamico.github.io/codes/.

[35] Peixoto TP . 2020. The graph-tool python library [accessed 2023 Oct]. https://graph-tool.skewed.de.

[36] Cao S, Lu W, Xu Q. 2016. Deep neural networks for learning graph representations. Proceedings of the AAAI Conference on Artificial Intelligence. Vol. 30. Palo Alto (CA): AAAI Press.

[37] Cao S, Lu W, Xu Q. Deep neural networks for learning graph representations [accessed 2023 Aug]. https://github.com/MdAsifKhan/DNGR-Keras.

[38] Zenodo . 2023. Repository with codes and data. Available on GitHub https://github.com/communityType/code and (an archived version) on Zenodo [accessed 2023 Oct] https://zenodo.org/doi/10.5281/zenodo.10053112.

[39] Newman MEJ, Leicht EA. 2007. Mixture models and exploratory analysis in networks. Proc Natl Acad Sci USA. 104(23):9564–9569. 17525150 10.1073/pnas.0610537104 PMC1887592

