# Supplementary material for: Nonassortative relationships between groups of nodes are typical in complex networks
Source: PNAS Nexus. 2023 Nov 6;2(11):pgad364. doi: 10.1093/pnasnexus/pgad364 (PMC10681970; doi:10.1093/pnasnexus/pgad364)
Supplement: pgad364_Supplementary_Data [file pgad364_supplementary_data.pdf]

Supplementary Materials for: Non-assortative relationships between groups of nodes are typical in complex networks PNAS Nexus, pgad364 (2023)

Cathy Xuanchi Liu,Tristram J. Alexander,Eduardo G. Altmann

November 27, 2023

S1 Data

We consider all networks from the Netzschleuder repository Ref. [30] in the main manuscript(accessed Aug. 2022) which belong to one of 5 common domains (Online Social, Economic, Technological, Biological, Informational) and satisfy the following constraints: 1) number of nodes in the largest connected component is between 50 and $20,000$; 2) not bipartite; and 3) not multilayer and no multiple types of edges. For the list of all networks see Table. S1.

In order to allow the systematic application of the different community-detection methods, we removed all self-loops and we consider only the largest connected component of the network. We found 52 networks with 26 undirected graphs and 26 directed graphs.

The data for our first case study in Sec. 4 is from Ref. [27][1] and corresponds to polblogs in the Netzschleuder repository. The data for the second case study in Sec. 4 was obtained from the online platform Twitter by (i) collecting all tweets containing the word 'climate' between November 2019 to June 2020, and coming from accounts with Australia specific locations in the user location metadata; (ii) identifying the top 100 most retweeted tweets each day during this period; (iii) capturing the user timelines for the authors of these daily top 100 most retweeted tweets; and (iv) keeping only retweets in these top-user timelines that retweet other top users. The resulting retweet network consists of 4,029 nodes representing the top users and 429,235 directed edges representing retweets of one top user by another top user (multi-edges are kept). The classification of these users was based on the manual classification of individual tweets performed in Ref. [28], where the classification of the tweet is attributed to the user. This data is available in our repository Ref. [33].

[1] Citations in this document correspond to the references listed at the end of the main manuscript.

**Type**	**Name**	**N**	**E**	**Information/Edge Direction**
Undirected	facebook_friends	329	1954	–
	ego_social/gplus_100329698645326486178	2211	79585	–
	facebook_organizations/L1	5793	45266	–
	facebook_organizations/L2	1429	32876	–
	facebook_organizations/M1	3862	87324	–
	facebook_organizations/M2	320	2369	–
	facebook_organizations/S1	165	726	–
	facebook_organizations/S2	5524	94218	–
	blumenau_drug	75	181	–
	celegans_metabolic	453	4574	–
	fullerene_structures/C1500	1500	2250	–
	human_brains/BNU10025864_1_DTI_AAL	116	1563	–
	interactome_pdz	161	209	–
	budapest_connectome/all_20k	1015	70654	–
	celegans_interactomes/wi2007	1108	1500	–
	collins_yeast	1004	8319	–
	interactome_vidal	2783	6007	–
	interactome_yeast	1458	1948	–
	kegg_metabolic/aae	880	2368	–
	tree-of-life/394	786	3874	–
	power	4941	6594	–
	route_views/19971108	3015	5156	–
	adjnoun	112	425	–
	polbooks	105	441	–
	wiki_science	677	6517	–
	bible_nouns	1707	9059	–
Directed	anybeat	12645	67053	Different
	qa_user/mathoverflow_c2a	13599	139534	Same
	inploid	14360	57101	Same
	faculty_hiring_us/domain_natural_sciences	2142	30381	Same
	faculty_hiring/business	113	8539	Same
	faculty_hiring/computer_science	206	4633	Same
	faculty_hiring/history	145	4347	Same
	un_migrations	232	11228	Same
	celegansneural	297	2359	Same
	cintestinalis	205	2854	Same
	foodweb_little_rock	183	2476	Same

	fresh_websAkatoreA	84	227	Same
	interactome_figeys	2217	6438	Different
	interactome_stelzl	1615	6075	Different
	messal_shale	700	6414	Different
	yeast_transcription	664	1066	Same
	ecoli_transcription/v1.0	329	456	Same
	celegans_2019/male_ chemical	559	5246	Same
	caida_as/20070917	8020	36406	Different
	gnutella/04	10876	39994	Different
	jung	6120	138706	Different
	software_dependencies/jdk	6434	150985	Different
	polblogs	1222	19086	Different
	webk/bwebkb_wisconsin_ link1	280	1106	Same
	word_adjacency/darwin	7377	46279	Same
	dblp_cite	12494	49687	Different

Table S1: Datasets Table: 26 undirected graphs and 26 directed graphs. Columns include network type(Type), network name(Name),node number(N), edge number(E) and the consistency between information flow and edge direction(Information/Edge Direction).

S2 Community detection methods

The five community-detection methods we apply to each network g were chosen to represent different approaches used in this area:

(i) Modularity-based methods search for partitions of g which maximize a quality function (the modularity) which favours groups of densely connected nodes. Here we use Louvain with the implementation available in Ref. [9,35].

(ii) Spectral approaches are based on spectra of matrices that represent the network and can often be seen as a minimization on the number of edges between groups. Here we use the version of spectral clustering based on the Bethe-Hessian matrix for heterogeneous graphs available at Ref. [31,38].

(iii) A probabilistic approach based on statistical inference can be obtained using Stochastic Block Models (SBM) as the random-graph generative process Ref. [24,25,26,34].Here we use the degree corrected SBM available in Ref.[36].

(iv) Dynamical processes that take place on networks provide an alternative approach to partition nodes into groups. We use the most popular approach, Infomap Ref. [11]., with the implementation available in Ref.[37].

(v) Deep-learning methods are increasingly being applied to the community-detection problem, in combination with a variety of approaches Ref. [5] Here we use the popular DNGR method for learning graph representations, as proposed in Ref. [32] and implemented in Ref. [39]. This method can be used to partition nodes into groups, as considered here, using an additional clustering method. We use k-means as in Ref. [32], with a specified number of communities. In the main text we show results for $B = 10$, similar results are obtained for different B's (see SM-Sec. S5 below).

The implementation of these five methods is available also in our repository Ref. [33].

S3 Community Structure Statistics

Consider a $B \times B$ edge density matrix $\boldsymbol{\omega}$ obtained applying a community-detection method to a network g. The $M_{B\times B}$ interaction classification matrix of $\boldsymbol{\omega}$ contains the community structure type τ of all pairs of communities $r, s \in [0, B]$, with $\tau \in$ {"Assortative", "Core-Periphery", "Disassortative", "Source-Basin"}, obtained using the classification introduced in Fig. 2 and Eqs. (3)-(4). The fraction of τ of a network g is then given by counting how often τ appears in M as

$$P_\tau = \frac{1}{B(B-1)} \sum_{r \neq s} \delta_{\tau, M_{r,s}}, \tag{1}$$

where $\delta_{x,y} = 1$ if $x = y$ and $\delta_{x,y} = 0$ if $x \neq 0$.

Knowing P_τ for all structure types τ in a network g, we define the dominant type τ_d as the τ with largest P_τ and occurring types τ_o as the set of τ's with $P_\tau > 0$. We then compute the *dominance* of each type τ over a set of networks $\boldsymbol{g}$ as the fraction of networks that has dominant type $\tau_d = \tau$ as

$$\text{Dominance}(\tau) = \frac{1}{|\boldsymbol{g}|} \sum_{g} \delta_{\tau, \tau_d}, \tag{2}$$

where $|\boldsymbol{g}|$ is the number of networks in $\boldsymbol{g}$. Similarly, the *occurrence* of type τ is computed as the fraction of networks in which τ occurs ($\tau \in \tau_o$) as

$$\text{Occurrence}(\tau) = \frac{1}{|\boldsymbol{g}|} \sum_{g} \sum_{\tau' \in \tau_o} \delta_{\tau, \tau'}. \tag{3}$$

In the results reported in this paper we applied the analysis above only to communities with more than 5 nodes, $N_r > 5$, because small communities play a minor role in explaining the properties of the network and the classification of their structure types τ is often not robust (when applying the tests reported in Sec. S4 below).

S4 Robustness Estimation

We test the statistical robustness of our classification of communities in types by (1) comparing to a null model and (2) considering small perturbations of the underlying network.

S4.1 Null model

We consider a null model of random density matrix $\boldsymbol{\omega}^{\text{null}}$ to assess the significance of the results we obtained applying our pairwise classification of community types to $\boldsymbol{\omega}$. Since our pairwise classification depends only on the ranking order of the entries in $\boldsymbol{\omega}$, we build $\boldsymbol{\omega}^{\text{null}}$ as a random $B \times B$ matrix with entries from 1 to B^2. We then apply our classification – defined in Fig. 2 of the manuscript – to obtain the interaction classification matrix M^{null}, the structure type fraction P_τ^{null} from Eqs. (5), and the dominant type τ_d^{null} and occurring types τ_o^{null} – from Eqs. (6) and (7) of the manuscript– for the null model. The result obtained from this analysis represent our random expectation for an arbitrary partition of the network g in B communities. We further propose a statistic over many networks $\boldsymbol{g}$ by generating null models for each g with the same B obtained in the original analysis. This method is summarized in Algorithm 1. The dominance and occurrence obtained using this null model are reported in Fig. 4 of the paper.

Algorithm 1 Density Null Model

Require: number of communities B of network g
1: Randomly assign ranking from 1 to B^2 to $\boldsymbol{\omega}_{B\times B}^{\text{null}}$
2: Compute M^{null} from the classification in Fig. 2
3: Calculate P_τ^{null} for community type τ from Eq. (S1)
4: Get dominant type τ_d^{null} and occurring types τ_o^{null}
5: Repeat the steps above for many networks $\boldsymbol{g}$ to compute Dominance$(\tau)^{\text{null}}$ and Occurrence$(\tau)^{\text{null}}$ from Eq. (S2) and (S3)

S4.2 Robustness of categorical classification

Our community structure classification is computed for a given partition of the network. In practice, small variations of the network or algorithms (e.g., the initialization of optimization steps) may result in (slightly) different partitions. To have a better understanding of the robustness of our classification and results to such variations, we introduce a bootstrapping method for estimating the uncertainty of the results obtained from our classification.

Given a certain graph g (with N nodes and E edges) and partition p with its corresponding pairwise community classification matrix M, we first rewire the graph by randomly choosing E edges (with replacement) from the edge list of g. We denote this generated graph g'. Then we compute the density matrix

Algorithm 2 Bootstrapping Robustness Estimation

Require: Graph g, partition p

Create a rewiring graph g'

Compute density matrix $\boldsymbol{\omega}'$

Get M' using classification in Eqs. (3) or (4)

Repeat above procedure k times

Calculate certainty P_{rs} from Eqs. (S4)

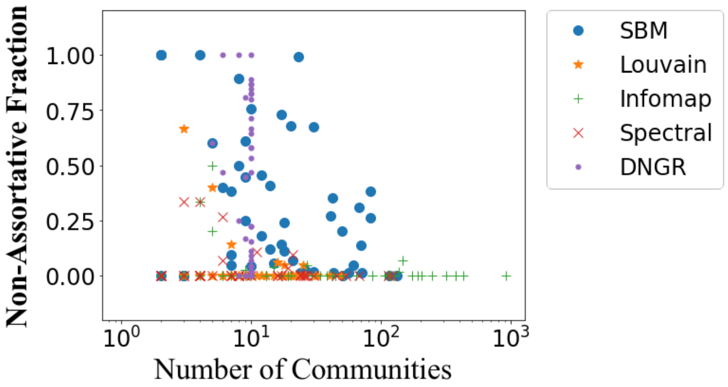

Fig. S1: Fraction of non-Assortative relationships (y-axis) as a function of the number of communities(x-axis) in empirical graphs. Each symbol represents a partition result of a network obtained using a specific community-detection method (see legend).

$\boldsymbol{\omega}'$ (based on g' and p) and we obtain a new interaction classification matrix M'. We repeat this procedure k times and compute the certainty for each pair of communities r and s, P_{rs}, as the fraction of all k interaction classifications that are equal to the original type

$$P_{rs} = \frac{1}{k} \sum_{1}^{k} \delta_{M_{rs}, M'_{rs}}. \tag{4}$$

We applied this analysis in several cases and confirmed that our main conclusions remain unchanged. In particular, for the two case studies reported in Sec. 4, we find that all classifications for $B = 5$ lead to $P_{rs} > 0.99$ except for one core-periphery classification in the blogs network which had $P_{r,s} = 0.86$ (see Fig. S2).

S5 Survey

Apart from dominance and occurrence shown in the main manuscript, we also consider an intermediate quantity – denoted proportional occurrence – to mea-

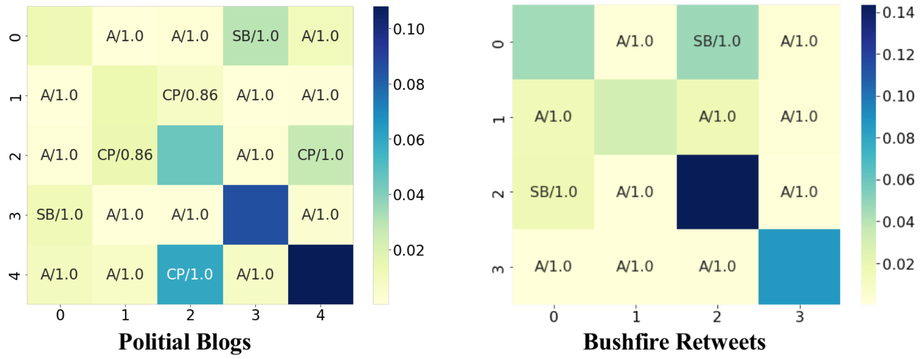

Fig. S2: Density matrices for the partition obtained by SBM with $B = 5$ for political blogs (left) and $B = 4$ for bushfire retweets(right). Colours indicate the community densities $\omega_{r,s}$. The blocks $b = 0, 1, \ldots, 4$ are ranked according to community size $N_r = \{354, 291, 291, 210, 76\}$(left) and $N_r = \{1473, 1031, 956, 563\}$(right) from community 0 to community 4(left)/3(right). As noted in the main text, the edge direction of the political blogs network is reversed when compared to the original dataset in order to fit the convention used in our text. The labels in each entry of the matrix correspond to the structure type τ and robustness score obtained from Eqs.(8) (see Sec. S4, Algorithm 2), where "A" stands for "Assortative", "CP" stands for "Core-Periphery", "D" stands for "Disassortative" and "SB" stands for "Source-Basin". Left: Community 0 and 3 are in a Source-Basin relationship. Communities 4 and 2 are in a Core-Periphery relationship and communities 2 and 1 are in a Core-Periphery relationship (i.e., community 2 is a periphery in respect to 4 and core in respect to 1). Right: Community 0,1,2 are climate change supporter groups. Community 0 and 2 are in a Source-Basin relationship.

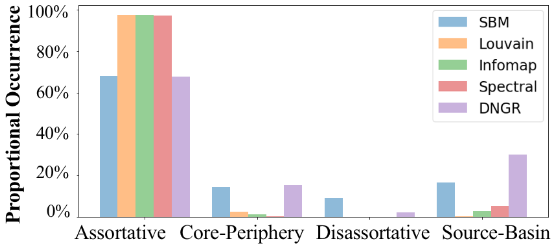

Fig. S3: Average fraction of pairs of the different types τ (Assortative, Core-Periphery, Disassortative, Source-Basin). For each network, we compute the proportional occurrence of type τ_i defined as Eq.(5). The reported results correspond to the average over 52 networks.

sure the classification result in empirical networks. Instead of focusing on the appearance of a type τ or in the most dominant type τ, here we focus on the fraction P_τ of pairs of a given type τ in each network. For each community structure type $\tau \in \{$"Assortative", "Core-Periphery", "Disassortative", "Source-Basin"$\}$, we average the fraction P_τ over a set of networks as

$$\text{Proportional Occurrence}(\tau) = \frac{1}{|\boldsymbol{g}|} \sum_{g} \sum_{\tau' \in \tau_o} P_{\tau'} \delta_{\tau,\tau'}. \quad (5)$$

This section shows additional analyses of our survey results. The results obtained in our survey using the proportional occurrence method are shown in Fig. S3. Separated results for directed and undirected networks are shown in Fig. S4. Finally, the results obtained using the DNGR method with different number of communities B are shown in Fig. S5.

References

The references cited in this document are listed in the main manuscript, PNAS Nexus, pgad364 (2023) `https://doi.org/10.1093/pnasnexus/pgad364`

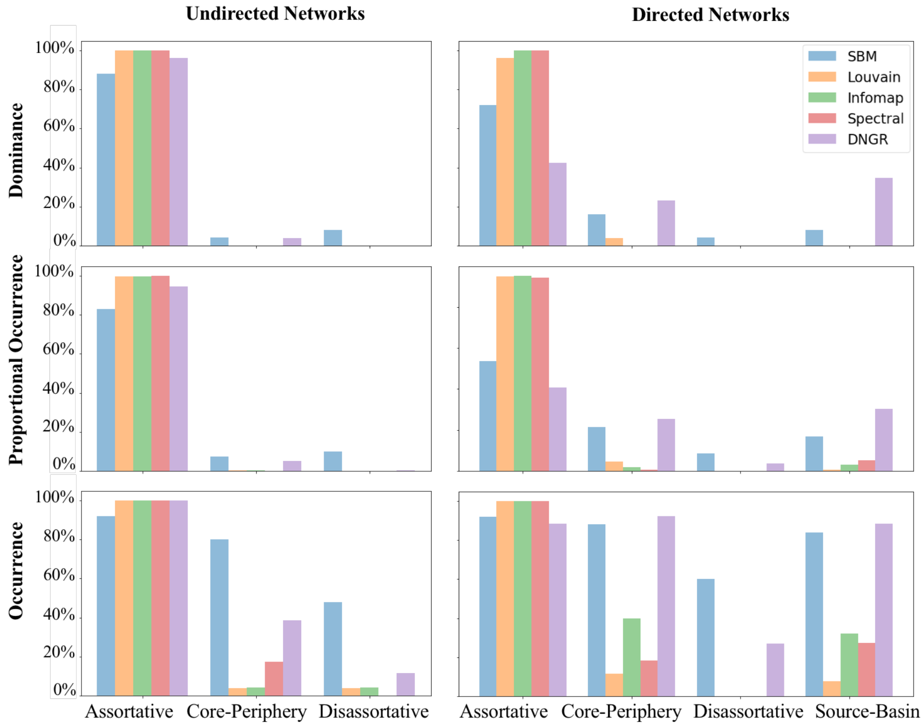

Fig. S4: Community structure type in empirical undirected graphs (left) and directed graphs (right).Top: fraction of networks (y-axis) in which community type (x-axis) is dominant. Middle: weighted fraction of networks in which community type appears. Bottom: fraction of networks in which community type appears.

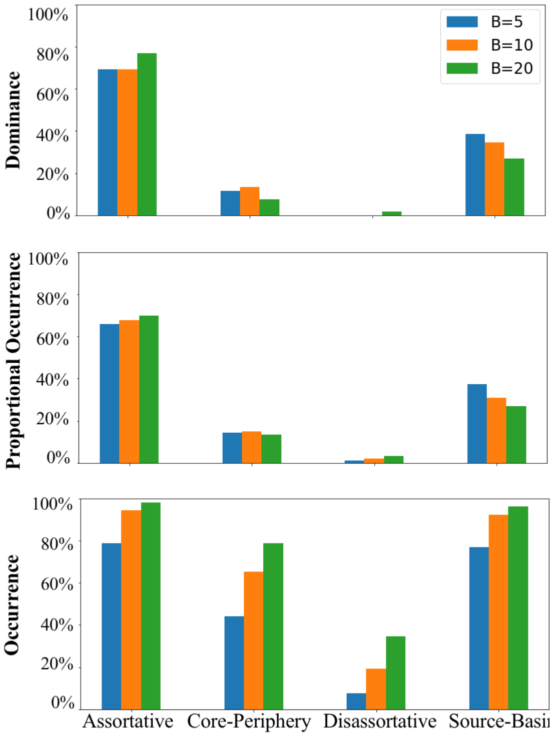

Fig. S5: Community structure type obtained applying the DNGR method to the empirical graphs in our survey. Results for three pre-defined number of communities are shown $B = 5, 10, 20$. Overall, the results with different B suggest an increasing occurrence of non-assortative structures and an increasing dominance (and proportional occurrence) of Assortative structure as B grows.
